# Minimum number of inertial measurement units needed to identify significant variations in walk patterns of overweight individuals walking on irregular surfaces

**DOI:** 10.1038/s41598-023-43428-9

**Published:** 2023-09-27

**Authors:** Tasriva Sikandar, Mohammad Fazle Rabbi, Kamarul Hawari Ghazali, Omar Altwijri, Mohammed Almijalli, Nizam Uddin Ahamed

**Affiliations:** 1 School of Health and Rehabilitation Sciences, The University of Queensland, Brisbane, QLD 4072 Australia; 2 School of Health Sciences and Social Work, Griffith University, Gold Coast, QLD 4222 Australia; 3 Faculty of Electrical and Electronics Engineering, University of Malaysia Pahang, 26600 Pekan, Malaysia; 4 Biomedical Technology Department, College of Applied Medical Sciences, King Saud University, Riyadh, Saudi Arabia; 5 School of Medicine, University of Pittsburgh, Pittsburgh, PA USA

**Keywords:** Health care, Biomedical engineering

## Abstract

Gait data collection from overweight individuals walking on irregular surfaces is a challenging task that can be addressed using inertial measurement unit (IMU) sensors. However, it is unclear how many IMUs are needed, particularly when body attachment locations are not standardized. In this study, we analysed data collected from six body locations, including the torso, upper and lower limbs, to determine which locations exhibit significant variation across different real-world irregular surfaces. We then used deep learning method to verify whether the IMU data recorded from the identified body locations could classify walk patterns across the surfaces. Our results revealed two combinations of body locations, including the thigh and shank (i.e., the left and right shank, and the right thigh and right shank), from which IMU data should be collected to accurately classify walking patterns over real-world irregular surfaces (with classification accuracies of 97.24 and 95.87%, respectively). Our findings suggest that the identified numbers and locations of IMUs could potentially reduce the amount of data recorded and processed to develop a fall prevention system for overweight individuals.

## Introduction

An irregular surface is a potential risk factor of falling while walking for all body weights^1^. In fact, half of the fall-related events (e.g., tripping, slipping, and stumbling) occur while walking on uneven or irregular surfaces such as paved and sloped surfaces and staircases^2–4^. In general, overweight (body-mass-index [BMI] > 25.0^5^) individuals, including young, middle-aged and older adults, are likely to fall more than normal-weight individuals^1,6–8^. In addition, falls in the overweight population (both male and female) are linked to an altered gait, postural instability, significant postural sway, and inability to adjust the corresponding walk pattern while walking on irregular surfaces^9–13^. Unfortunately, the characterization of the walk patterns of the overweight population in a real-world scenario is a challenging task due to the limited number of suitable devices for recording gait data. In this scenario, wearable sensors such as an inertial measurement unit (IMU) could be an advantage for recording gait data from overweight individuals to analyze their walk patterns in a range of irregular surfaces^14,15^. A comprehensive analysis of walk patterns defined by IMU data may help us better explain the underlying mechanism of falling because IMU data are able to reveal the kinematic variability during walking in real-world irregular surfaces^16–18^. However, the number and location of IMUs are two important metrics that might restrict the data recording and subsequent application of IMUs for fall prevention.

The identification of appropriate body locations (e.g., upper limbs, lower limbs and torso) for IMUs is needed because using the minimum number of wearable devices is preferred by individuals across different ages^19,20^. One approach to identify the minimum number of IMUs is exploring the IMU data recorded from a combination of body locations and then using statistical analysis and/or artificial intelligence (AI)-based methods (e.g., deep learning method) to identify the IMU data with the greatest variability^21,22^. The rationale for this study is that changes in IMU data would represent the underlying variation of walk patterns across surfaces and could potentially identify a set of significant body locations for the attachment of IMUs^4,23,24^. Subsequently, IMUs attached to body locations that capture invariable walk patterns might be abandoned. Although previous studies^9,16,17^ explored IMU data from different body locations (e.g. chest, hip, thigh and shank) of overweight individuals, those did not consider real-world scenarios but rather clinical settings with conventional walking surface (e.g., treadmill flat surface). However, the walk patterns on sloped and inclined surfaces (e.g., staircase) showed more variability than those on flat surfaces (e.g., even and uneven)^4,23^. Therefore, IMU attached to a single body location might not provide data with the highest variability for a range of surfaces that necessitates more exploration of the combination of both body location and irregular surfaces.

In addition to a statistical analysis, a deep learning method could be used to identify the most appropriate body locations in the upper and lower limbs^21^. While exploring the variation of IMU data from different body locations, a deep learning method could be employed to use the IMU data to assess whether the walk patterns could be classified. Intuitively, the body locations identified by the statistical analysis should be the best locations identified with the deep learning method because these body locations provide the IMU data with the greatest variability, but this hypothesis remains to be explored. Although previous studies^4,23,24^ have investigated deep learning-based walk-pattern classification using data from a limited number of body locations in the upper and lower limbs, not all possible combinations of body locations (in e.g., upper limbs, lower limbs, and torso) were studied. Potentially, the statistical analysis and AI-based deep learning classification of the walk patterns of overweight individuals using all possible combinations of body locations may help identify the combination(s) of body location corresponding to a user’s preference regarding their minimum IMU(s) count and body locations^19,20,25^. To the best of our knowledge, a statistical analysis of the variation in IMU data and a deep learning-based classification of walk patterns using IMU data from all possible combinations of body locations while walking on real-world irregular surfaces have not been previously performed.

The objective of this study was to identify the minimum number of body locations for the attachment of IMUs to obtain data that could best represent the walk patterns of the overweight population in real-world irregular surfaces. This study investigated the hypothesis that more than one IMU are needed to classify the walk patterns of overweight individuals while walking on various irregular walking surfaces individually with a correct classification rate (i.e., CCR) greater than 90%. The findings of this study will contribute to the development of AI-based healthcare applications for wearable devices that could recognize a range of walking surfaces with creditable performance and could be attached at user-friendly body locations to reduce the risk of potential falls among the overweight population.

## Methods

In this study, human walk data from a publicly available dataset available in Scientific Data^26^ were used, and the dataset study was approved by the Harvard and Northeastern Institutional Review Boards (Boston, MA, USA). Short but mandatory descriptions of the participants and data collection procedures have been included in the following sections.

### Participants

IMU data were collected from ten overweight participants (eight males and two females) aged 24.78 ± 5.14 years with a height of 173.43 ± 4.99 cm, a weight of 84.68 ± 9.5 kg, and a BMI of 28.47 ± 3.38 while walking on irregular surfaces. The BMI of the participants (> 25.0) was classified according to the World Health Organization’s (WHO’s) guidelines that included both overweight and obese populations^5^. The participants were among the sedentary population who lived in proximity to an urban US campus. The participants were healthy adults with no reported neurological or musculoskeletal conditions that affect their gait or posture, no lower limb injuries, and no history of fall injuries in last two years.

### Data collection

**Figure 1:**
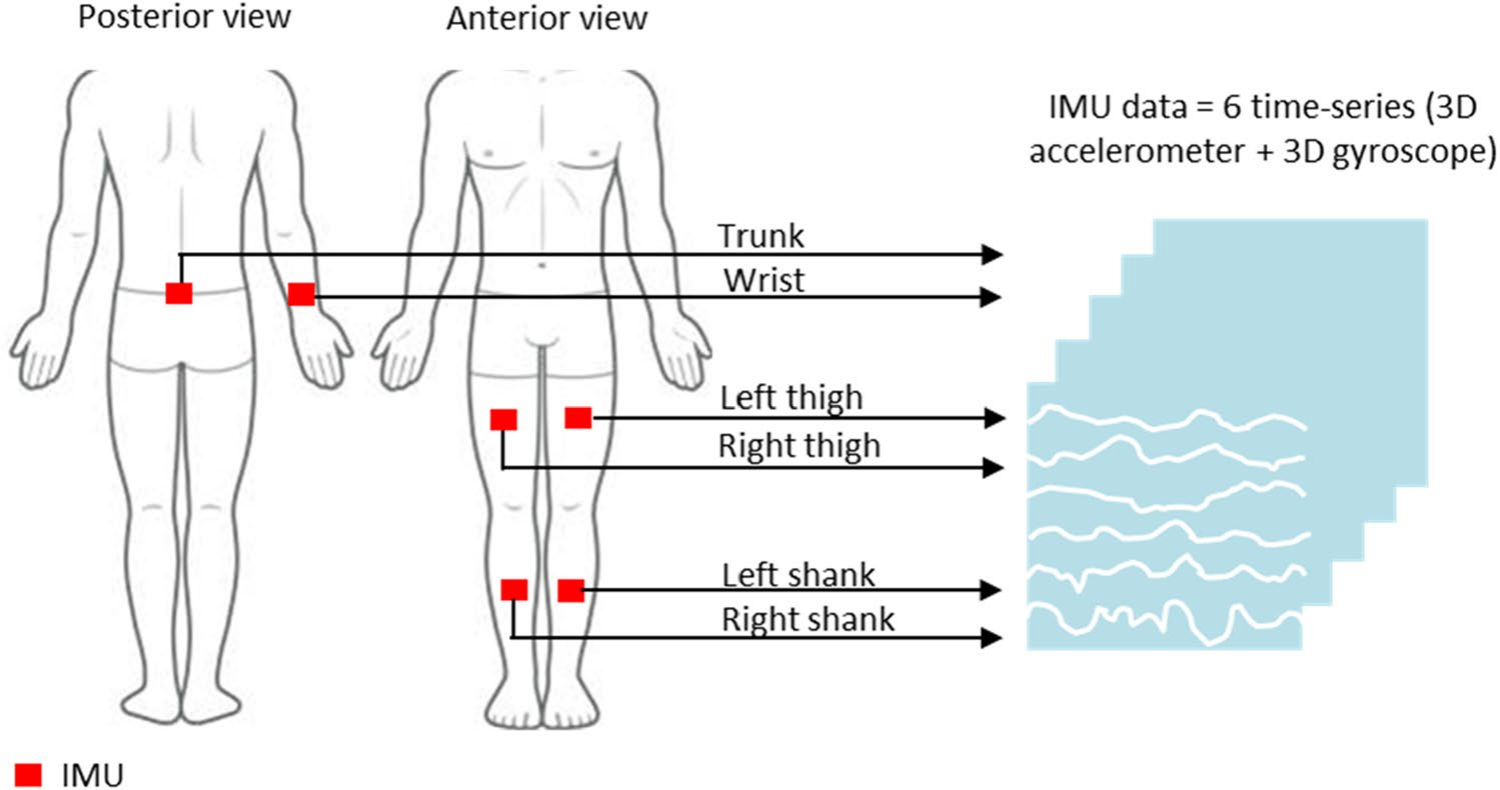
Placement of IMUs on six body locations. Acceleration (m/s^2^) in the vertical, mediolateral, and anterior–posterior directions is denoted 3D accelerometer data. The rate of turn (rad/s) in the vertical, mediolateral, and anterior–posterior directions is defined as 3D gyroscope data. IMU—inertial measurement unit; m/s^2^—meter/second^2^; 3D—three dimensional; rad/s—radian/second.

The entire data collection procedure has been broadly discussed previously^26^. The current study only used three-dimensional (3D) accelerometer and gyroscope data generated from six IMUs (MTw Awinda, Xsens, Enschede, Netherlands) attached to six locations in the body of each participant: trunk (fifth lumbar vertebra), right wrist (dorsal), left and right thigh (bilateral anterior), and left and right shank (bilateral anterior) (Fig. 1). The 3D accelerometer data consisted of acceleration (m/s^2^), whereas the 3D gyroscope data consisted of the rate of turn (rad/s) in the vertical, mediolateral and anterior–posterior directions (Fig. 1). Six walking trials were performed over seven different surfaces: flat uneven (horizontal, cobble stone [26 × 18 cm blocks] paved), banked left (uneven, left cross-slope and cement), banked right (uneven, right cross-slope and cement), stair up (sloped, step, cement and upward), stair down (sloped, step, cement and downward), slope up (sloped, straight, cement and upward), and slope down (sloped, straight, cement and downward). During each walking trial, the participants walked at their self-preferred speed (~ 1.5 m/s) and swung their arms spontaneously. For each walking trial of each participant, 36 time-series IMU data spanning 16.4 ± 4.2 s (i.e., 6 IMUs × [3D accelerometer + 3D gyroscope] data) from six body locations (Fig. 1) were recorded with a sampling frequency of 100 Hz. Furthermore, all the data were smoothed using 2nd-order Butterworth low-pass filter with a cut-off frequency of 6 Hz^26^.

### Data processing, walk pattern creation, and augmentation

In this study, the first 4 s of IMU data (i.e., 400 samples of 6 time-series [3D accelerometer + 3D gyroscope] data from each IMU) from each participants were used for the analysis; these data duration was comparable to the time duration used in previous studies^4^. A statistical analysis was then performed to assess the variation in IMU data across six body locations and across seven walking surfaces. The statistical analysis process is described in Section “Statistical analysis of IMU data”.

**Figure 2:**
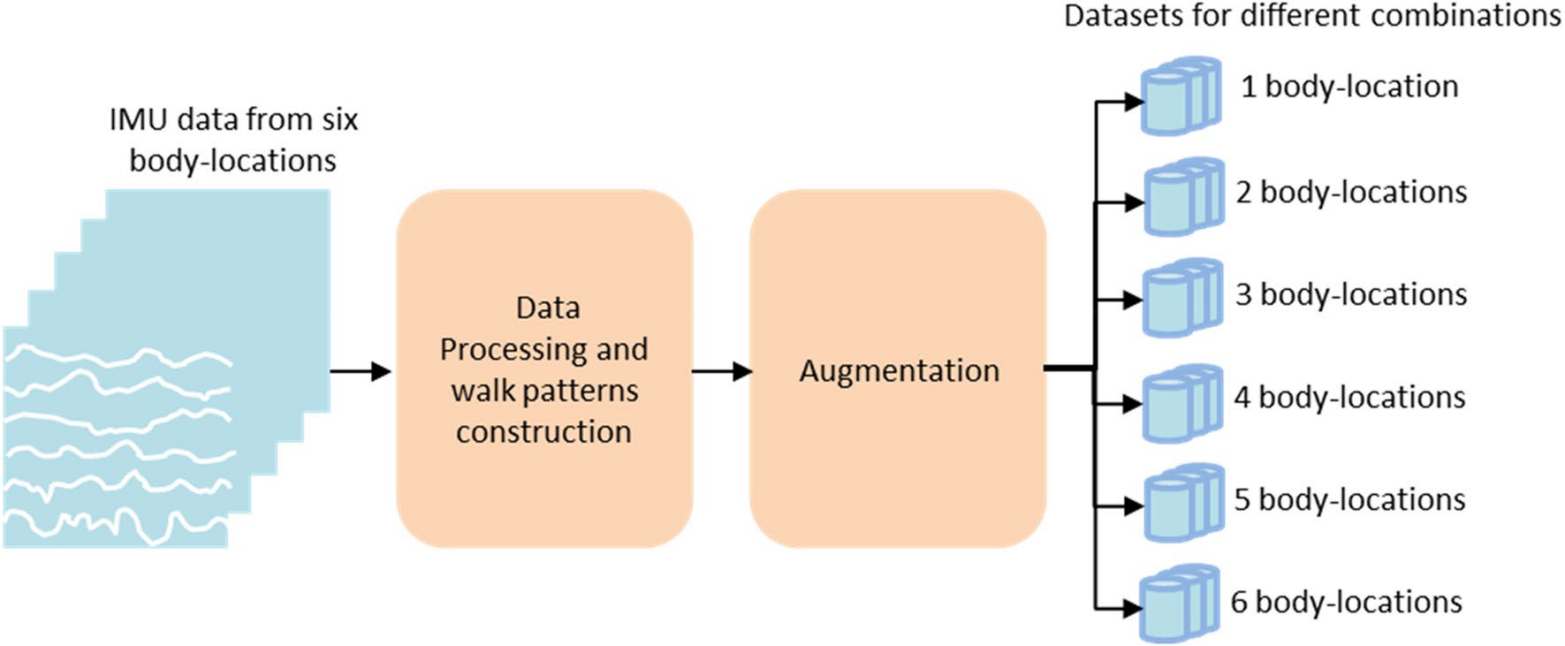
Creation of walk patterns using IMU data collected from six body locations and structuring of the datasets of the walk patterns from different combinations of body locations.

After the statistical analysis, the walk patterns with dimensions of (6 × 400), (12 × 400), (18 × 400), (24 × 400), (30 × 400) and (36 × 400) (i.e., number of body location(s) × IMU data with 400 samples) were constructed using the IMU data from each of the six body location(s) of each participant over seven different surfaces, respectively. The walk patterns from the different combinations of body locations were arranged according to Eq. (1):

1 $$C\left( n \right) = \frac{6!}{{n!\left( {6 - n} \right)!}},n = 1,2,3,...6$$

where $C(n)$ is the number of combinations of the included body locations, *n* is the number of body locations included in the combination and (6-*n*) is the number of body locations not included in the combination^22^. Thus, a total of 63 combinations of body locations (full list available in Section “Combinations of body location”) were produced to create walk patterns. For each combination of body locations, a dataset of walk patterns over the seven different surfaces were created (Fig. 2 and Table 1). Each dataset consisted of 60 walk patterns (i.e., 6 walk trials on a surface × 10 participants) for each surface, which corresponded to a total of 420 walk patterns for the seven surfaces. All walk patterns in each dataset were augmented 10 times with random rotations around the participant’s IMU axes to simulate the variation in the walk patterns due to variation in the IMU sensor orientation^25,27^. After this augmentation, each dataset contained 600 walk patterns for a particular surface and a total of 4200 walk patterns for the seven surfaces. Finally, a deep learning-based method was utilized to classify the walk patterns in the different datasets according to the walking surfaces. The process used for the deep learning-based classification of walk patterns in different surfaces is described in Section “Classification of walk patterns in different surfaces”.

**Table 1:** Description of the walk patterns in the different datasets.

No. of body-locations	Dimensions of walk-pattern	No. of datasets constructed for body-locations combination (Eq. 1)	No. of walk-patterns/dataset (before augmentation)	No. of walk-patterns/dataset (after augmentation)
1	6 × 400 [(3D accelerometer + 3D gyroscope) × 1 IMU] × 400	6	420 (60 walk-patterns × 7 surfaces)	4200 (600 walk-patterns × 7 surfaces)
2	12 × 400 [(3D accelerometer + 3D gyroscope) × 2 IMU] × 400	15	420 (60 walk-patterns × 7 surfaces)	4200 (600 walk-patterns × 7 surfaces)
3	18 × 400 [(3D accelerometer + 3D gyroscope) × 3 IMU] × 400	20	420 (60 walk-patterns × 7 surfaces)	4200 (600 walk-patterns × 7 surfaces)
4	24 × 400 [(3D accelerometer + 3D gyroscope) × 4 IMU] × 400	15	420 (60 walk-patterns × 7 surfaces)	4200 (600 walk-patterns × 8 surfaces)
5	30 × 400 [(3D accelerometer + 3D gyroscope) × 5 IMU] × 400	6	420 (60 walk-patterns × 7 surfaces)	4200 (600 walk-patterns × 7 surfaces)
6	36 × 400 [(3D accelerometer + 3D gyroscope) × 6 IMU] × 400	1	4200 (600 walk-patterns × 7 surfaces)	

### Statistical analysis of IMU data

A statistical analysis of the IMU data from each of the six body locations of all participants was performed to evaluate the significant differences across (i) the seven walking surfaces based on the same body location and (ii) the six body locations over the same walking surface (Table 2). All statistical comparisons in this study were performed using Welch’s one-way repeated-measures analysis of variance (ANOVA) with Bonferroni correction (*p* < 0.001) post hoc analysis. Prior to ANOVA, the Shapiro–Wilk test for normality of the data distribution, Levene’s homoscedasticity test, Mauchly’s sphericity test and Greenhouse–Geisser correction were assessed. All statistical analyses in this study were performed using IBM SPSS Statistics v26 software (IBM Corp, Armonk, NY, USA). In Section “Findings from the statistical analysis”, the results from the statistical analysis are presented by assigning the terms “Significant” (i.e., *p* < 0.001) and “Insignificant” (i.e., *p* > 0.001) to the differences in the IMU data from the same body location across the different walking surfaces and across the different body locations over the same walking surface.

**Table 2:** Description of the statistical comparisons performed (i) across the different walking surfaces using data from the same body location and (ii) across different body locations over the same walking surface.

Statistical analysis	Comparisons performed on data of overweight group	Total comparisons performed
Across surfaces within same body-location	Flat uneven vs banked left, flat uneven vs banked right, flat uneven vs stair up, flat uneven vs stair down, flat uneven vs slope up, flat uneven vs slope down, banked left vs banked right, banked left vs stair up, banked left vs stair down, banked left vs slope up, banked left vs slope down, banked right vs stair up, banked right vs stair down, banked right vs slope up, banked right vs slope down, stair up vs stair down, stair up vs slope up, stair up vs slope down, stair down vs slope up, stair down vs slope down, slope up vs slope down— have been performed on six outputs (i.e. 3D accelerometer and 3D gyroscope) of each of the six IMU sensors attached to body-locations (trunk, wrist, left thigh, right thigh, left shank and right shank)	756
Across body-locations over same surface	Trunk vs wrist, trunk vs left thigh, trunk vs right thigh, trunk vs left shank, trunk vs right shank, wrist vs left thigh, wrist vs, right thigh, wrist vs left shank, wrist vs right shank, left shank vs left thigh, left shank vs right thigh, right shank vs right thigh, right shank vs left thigh, left shank vs right shank and left thigh vs right thigh— have been performed on six outputs (i.e., 3D accelerometer and 3D gyroscope) of each of the six IMU sensors for each of the seven surface (flat uneven, banked left, banked right, stair up, stair down, slope up and slope down)	630

### Classification of walk patterns in different surfaces

**Figure 3:**
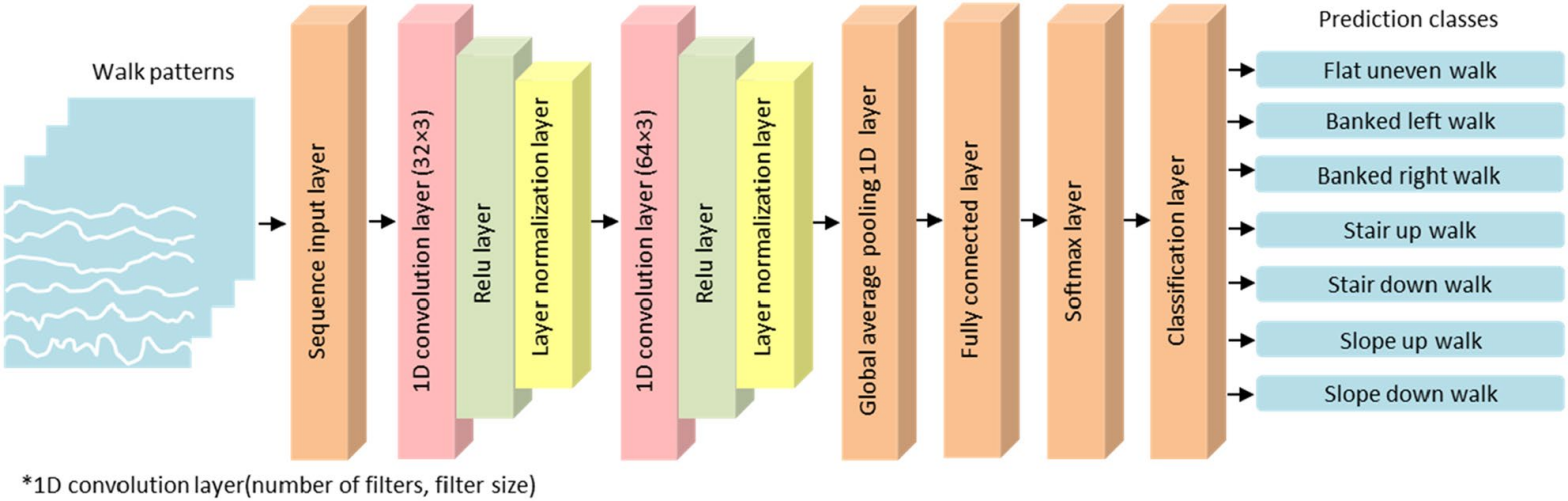
Outline of the network structure.

The current study utilized Convolution Neural Network (CNN)-based deep learning networks to classify the walk patterns of the overweight population on different surfaces using each dataset described in Table 1. The CNN-based deep learning network starts with a sequence input layer (Fig. 3). According to the size of the walk patterns (i.e., 6 × 400, 12 × 400, 18 × 400, 24 × 400, 30 × 400 and 36 × 400, respectively) in the datasets, the size of the sequence input layer varies between 6, 12, 18, 24, 30, and 36. The input layer is followed by the following structure: (32 × 3) size 1D convolution layer with casual padding + relu layer + normalization layer. Another structure [(64 × 3) size 1D convolution with casual padding + relu layer + layer normalization layer] was also used. A global average pooling 1D layer, a fully connected layer with seven outputs specifying the seven classes, a softmax layer and a classification layer with cross-entropy function then completed the network^28–33^. Here, the seven classes of the fully connected layer specify the walk patterns of the overweight population on seven surfaces (i.e., flat uneven, banked left, banked right, stair up, stair down, slope up and slope down). The other properties of the abovementioned layers were selected according to the default values using MATLAB 2021b (MATLAB™, Natick, MA, USA). Previous research has shown that this depth set-up of the CNN is sufficient for dimensionality reduction, feature extraction, and local connectivity extraction and for obtaining non-overfitted and high-accuracy solutions to similar classification problems^4, 28^.

To ensure that the classification approach was robust and that the data were not over-fitted, the performance of the developed CNN-based deep learning network was evaluated using the 10-fold cross-validation method with a total of 90 combinations of the training, validation and testing multiclass subsamples. The training, testing and validation subsamples, which consisted of 80, 10 and 10% of the walk-patterns, respectively, comprised seven classes in each fold. The training of the networks using 10-fold cross-validation was conducted using Adam optimizer with the following parameters: initial learning rate = 0.001, decay rate of squared gradient moving average = 0.99, gradient threshold method = ‘global-12norm’, gradient threshold = 0.9, epochs = 500, batch size = 64, data shuffling = ‘never’. MATLAB 2021b (MATLAB™, Natick, MA, USA) software with an Intel(R) Core (TM)i5-2400CPU, 3.10-GHz computer was used for model training and for validation and testing of the datasets.

The mean(± standard deviation [SD]) classification accuracy (CA) achieved with the CNN-based deep learning networks using walk patterns from different body location combinations was compared in Section “Findings from the classification of walk patterns using data from different body location combinations”. In addition, the results from the classification of the walk patterns on different surfaces using different body location combination(s) were evaluated using a confusion matrix and are presented in terms of the mean(± SD) correct classification rate (CCR) compared among different body location combination(s) and different surfaces. Furthermore, Section “Findings from the classification of walk patterns using data from different body location combinations” summarizes the body location combination(s) for which a CCR greater than 90% was achieved for the different walking surfaces.

## Results

### Findings from the statistical analysis

**Figure 4:**
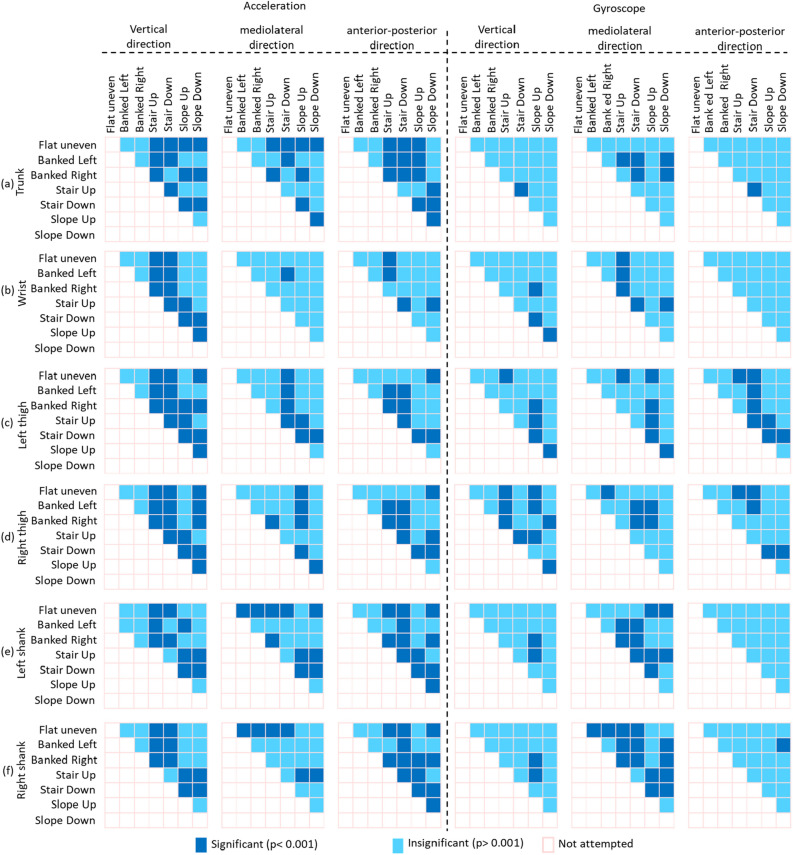
Summary of significant differences in IMU data (3D accelerometer and 3D gyroscope data) identified by statistical comparisons across seven walking surfaces using data from the (**a**) trunk (**b**) wrist (**c**) left thigh (**d**) right thigh (**e**) left shank and (**f**) right shank.

**Figure 5:**
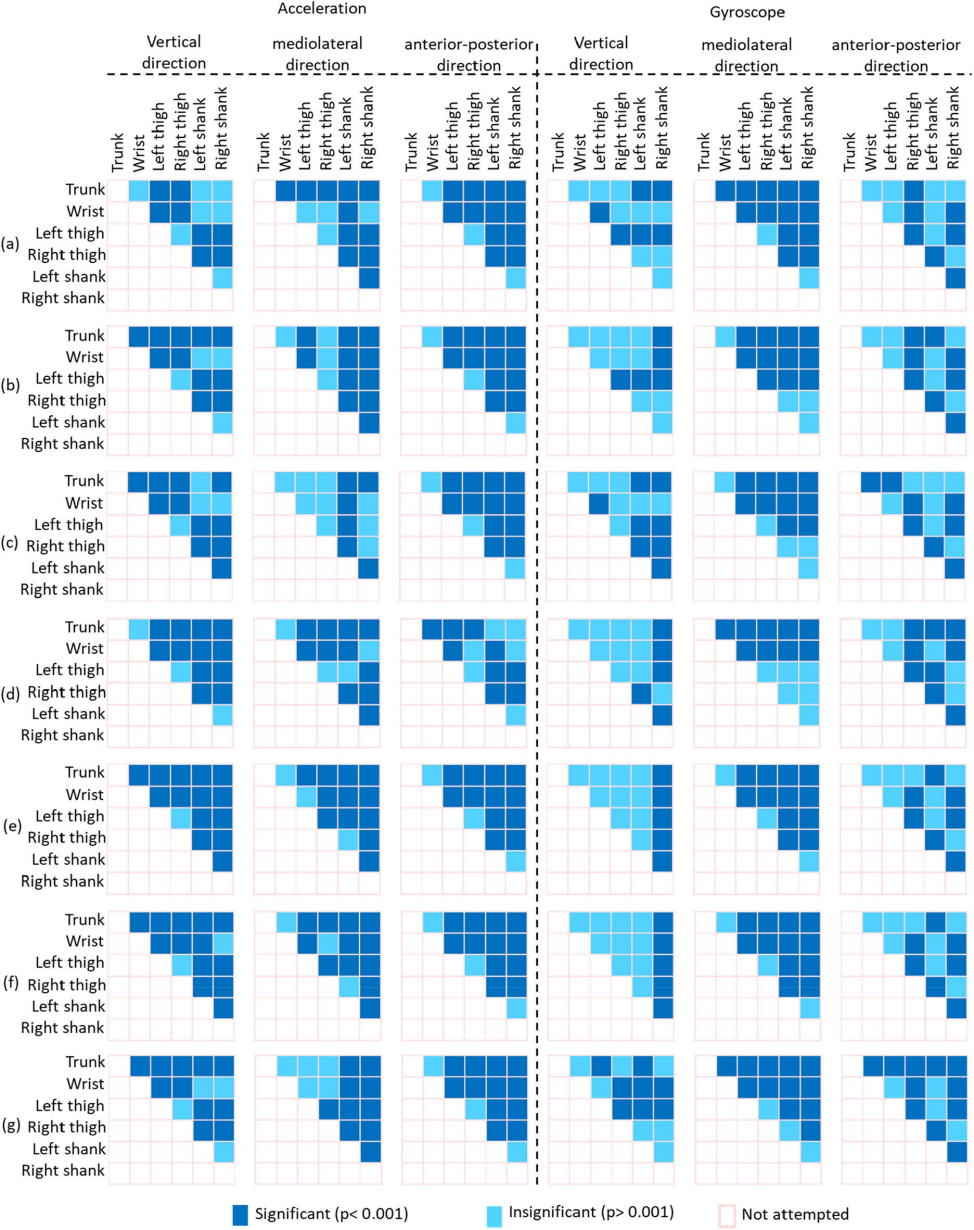
Summary of significant differences in IMU data (3D accelerometer and 3D gyroscope data) identified from statistical comparisons across the different body locations over the following surfaces: (**a**) flat uneven (**b**) banked left (**c**) banked right (**d**) stair up (**e**) stair down (**f**) slope up and (**g**) slope down.

Figures 4 and 5 show a summary of the significant differences in IMU data (i.e., 3D accelerometer and 3D gyroscope data in the vertical, mediolateral and anterior–posterior directions) identified from the statistical comparisons across (i) the seven different walking surfaces using data from the same body location, and (ii) the six different body locations over the same walking surface. The comparisons of IMU data across different walking surfaces (Fig. 4) showed that the differences in the 3D accelerometer data were more significant (*p* < 0.001) than those in the 3D gyroscope data. Moreover, the comparison across walking surfaces showed that the IMU data from the trunk, left thigh, right thigh, left shank and right shank exhibited more significant differences (*p* < 0.001) than those from the wrist (*p* > 0.001). Additionally, across all seven surfaces the IMU data from individual body locations did not reveal significant differences (*p* > 0.001). In contrast, comparisons of IMU data across the body locations (Fig. 5) showed that the differences in the 3D accelerometer data and those in the 3D gyroscope data exhibited appreciable significance (*p* > 0.001). Furthermore, the comparison of IMU data from different body locations revealed significant differences (*p* > 0.001) on a particular surface.

### Combinations of body location

Table 3 presents list of all possible combinations of six body locations determined by using Eq. 1. Combination number (comb. num.) in the table indicates each combination of body locations and thereby will be referred to as the individual combination in the rest of the results.

**Table 3:** All possible combinations of six body locations to attach IMU.

Comb. Num.	Body location	Comb. Num.	Body location	Comb. Num.	Body location
1	Trunk	22	Trunk, Left thigh, Right thigh	43	Trunk, Right thigh, Left shank, Right shank
2	Wrist	23	Trunk, Left shank, Right shank	44	Trunk, Left shank, Left thigh, Right thigh
3	Left thigh	24	Trunk, Left shank, Right thigh	45	Trunk, Right shank, Left thigh, Right thigh
4	Right thigh	25	Trunk, Left shank, Left thigh	46	Trunk, Wrist, Left shank, Right shank
5	Left shank	26	Trunk, Right shank, Right thigh	47	Trunk, Wrist, Left thigh, Right thigh
6	Right shank	27	Trunk, Right shank, Left thigh	48	Trunk, Wrist, Left thigh, Left shank
7	Trunk, Left thigh	28	Trunk, Wrist, Left thigh	49	Trunk, Wrist, Left thigh, Right shank
8	Trunk, Right thigh	29	Trunk, Wrist, Right thigh	50	Trunk, Wrist, Right thigh, Left shank
9	Trunk, Left shank	30	Trunk, Wrist, Left shank	51	Trunk, Wrist, Right thigh, Right shank
10	Trunk, Right shank	31	Trunk, Wrist, Right shank	52	Wrist, Left thigh, Left shank, Right shank
11	Trunk, Wrist	32	Wrist, Left thigh, Right thigh	53	Wrist, Right thigh, Left shank, Right shank
12	Wrist, Left thigh	33	Wrist, Left shank, Right shank	54	Wrist, Left shank, Left thigh, Right thigh
13	Wrist, Right thigh	34	Wrist, Left shank, Right thigh	55	Wrist, Right shank, Left thigh, Right thigh
14	Wrist, Left shank	35	Wrist, Left shank, Left thigh	56	Left shank, Right shank, Left thigh, Right thigh
15	Wrist, Right shank	36	Wrist, Right shank, Right thigh	57	Trunk, Left thigh, Right thigh, Left shank, Right shank
16	Left thigh, Right thigh	37	Wrist, Right shank, Left thigh	58	Wrist, Left thigh, Right thigh, Left shank, Right shank
17	Left thigh, Left shank	38	Left thigh, Left shank, Right shank	59	Trunk, Wrist, Left thigh, Right thigh, Left shank
18	Left thigh, Right shank	39	Right thigh, Left shank, Right shank	60	Trunk, Wrist, Left thigh, Right thigh, Right shank
19	Right thigh, Left shank	40	Left shank, Left thigh, Right thigh	61	Trunk, Wrist, Left thigh, Left shank, Right shank
20	Right thigh, Right shank	41	Right shank, Left thigh, Right thigh	62	Trunk, Wrist, Right thigh, Left shank, Right shank
21	Left shank, Right shank	42	Trunk, Left thigh, Left shank, Right shank	63	Trunk, Wrist, Left thigh, Right thigh, Left shank, Right shank

### Findings from the classification of walk patterns using data from different body location combinations

**Figure 6:**
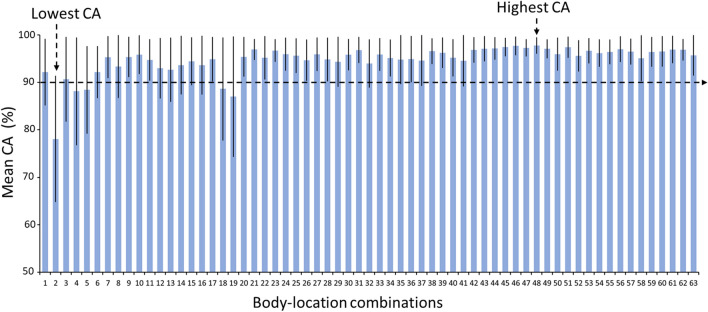
Comparisons of the mean CA achieved with the CNN-based deep learning networks using walk patterns from different body location combinations. CA—classification accuracy; the error bars represent standard deviations; body-location combinations numbers in x-axis represent individual combination listed in Table 3. Lowest CA achieving body-location combination—Wrist; highest CA achieving body-location combination—Trunk, Wrist, Left thigh, Left shank.

**Figure 7:**
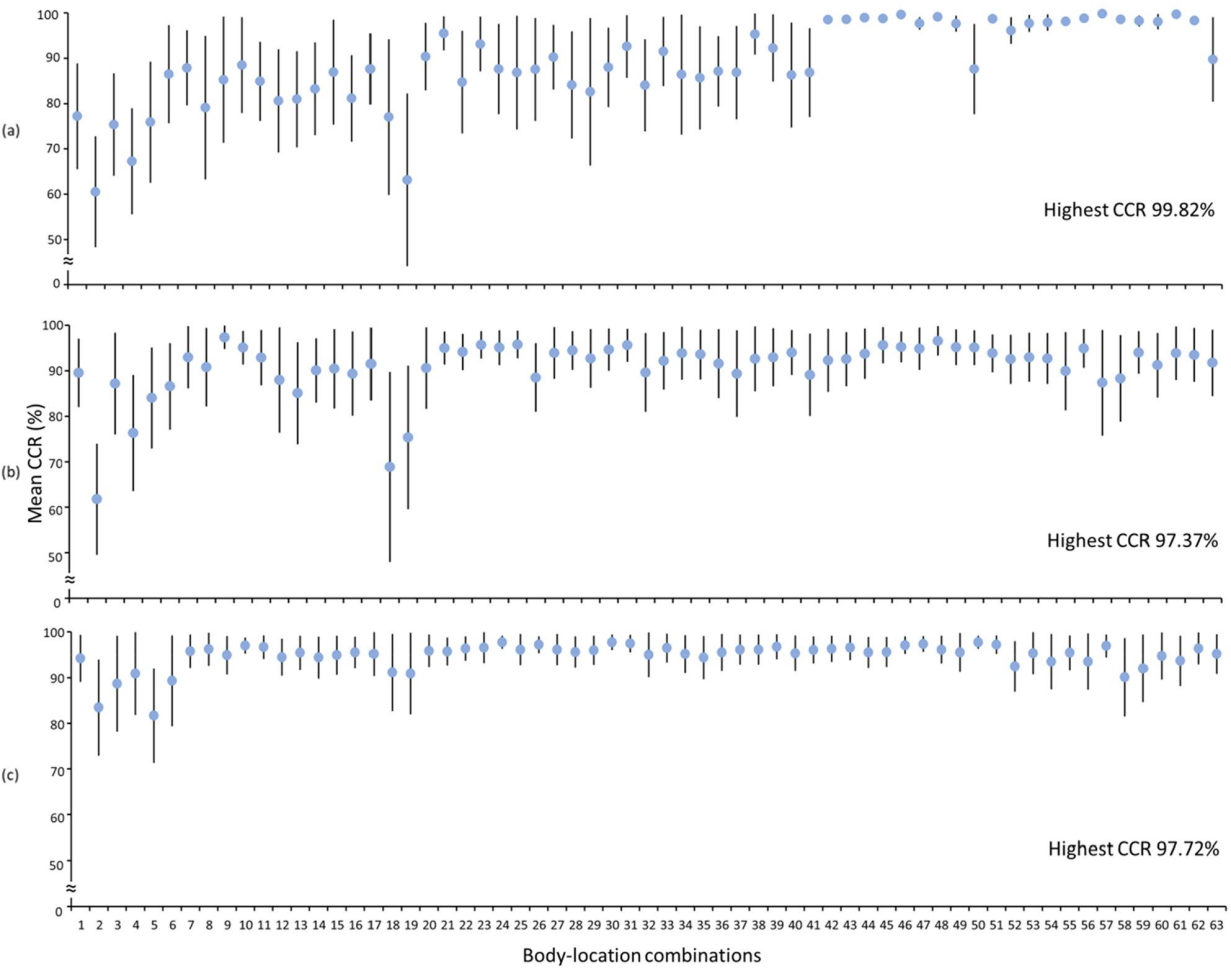
Comparison of the mean CCR from different body location combinations while walking on (**a**) flat uneven (**b**) banked left and (**c**) banked right surfaces. CCR—correct classification rate; the error bars represent standard deviations; body-location combinations numbers in x-axis represent individual combination listed in Table 3.

**Figure 8:**
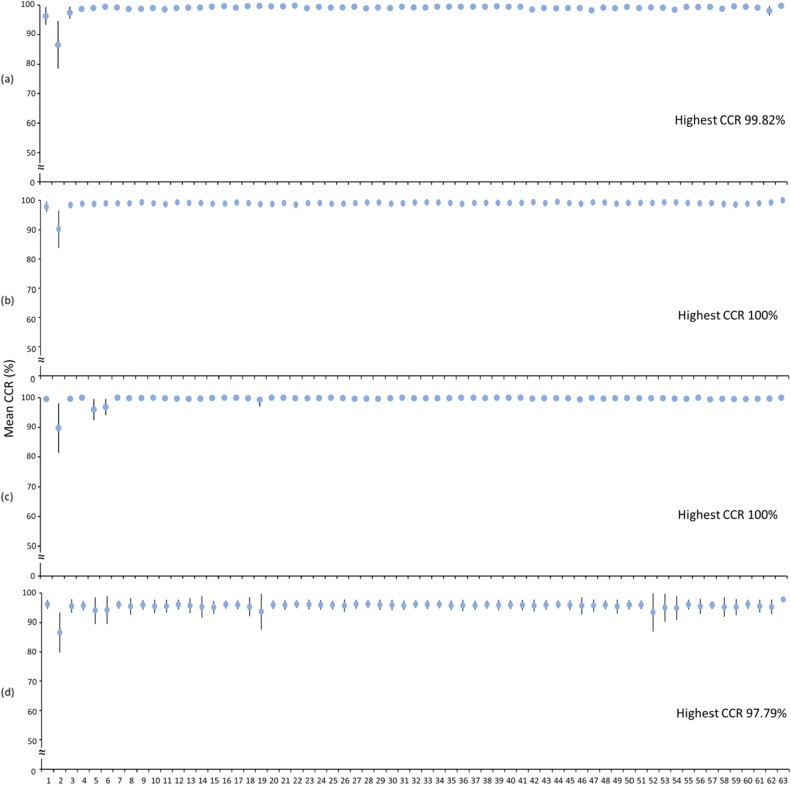
Comparison of the mean CCR from different body location combinations while walking on (**a**) stair up (**b**) stair down (**c**) slope up and (**d**) slope down surfaces. CCR—correct classification rate; the error bars represent standard deviations; body-location combinations numbers in x-axis represent individual combination listed in Table 3.

Figure 6 compares the CA (mean (± SD)) achieved with the CNN-based deep learning networks among walk patterns from different body location combinations. A combination of four body locations (i.e., trunk, wrist, left thigh and left shank) yielded walk patterns that achieved the highest CA (mean (± SD)) of 97.78 (± 1.72)%. In addition, the walk patterns from almost all body location combinations with the exception of the following achieved a mean CA higher than 90%: wrist and right/left shank. Further, comparisons of the CCR (mean (± SD)) among different body location combinations for the seven surfaces obtained from the CNN-based walk pattern classification are illustrated in Figs. 7 and 8. Walk patterns from a combination of multiple body locations achieved the highest CCR values of 99.82, 97.37, 97.72, 99.82, 100 and 97.79% for flat uneven, banked left, banked right, stair up, stair down and slope down surfaces, respectively, whereas only one body location achieved the highest CCR (i.e., 100%) for the slope up surface (Figs. 7 and 8).

**Figure 9:**
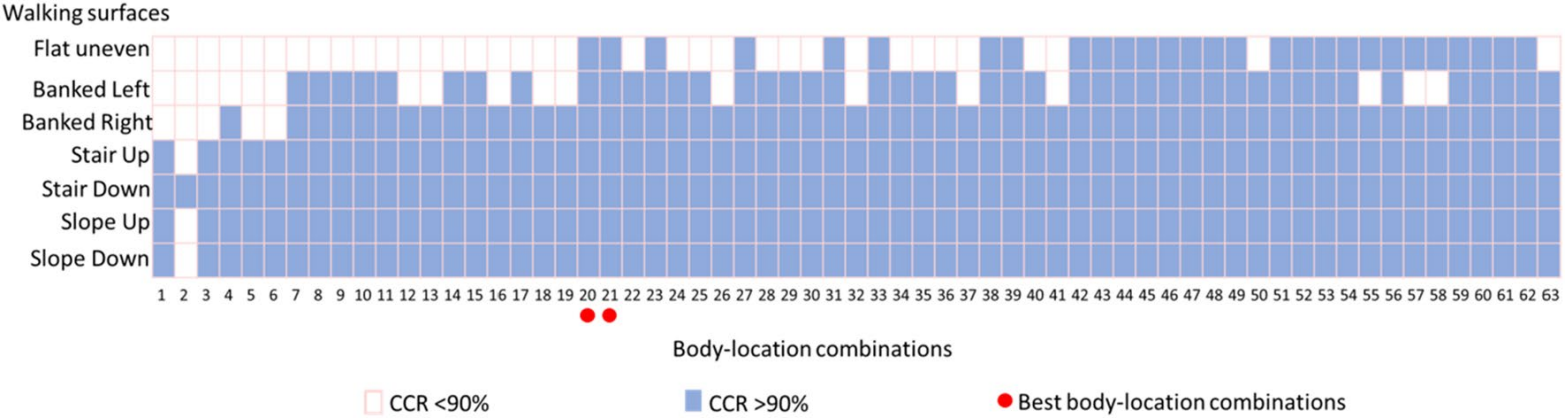
Summary of body location combinations that achieved a CCR greater than 90% for different walking surfaces and classification performance of the best body location combinations. CCR—correct classification rate; body-location combinations numbers in horizontal axis represent individual combination listed in Table 3. Two best body-location combinations are the right shank and left shank and the right thigh and right shank.

Figure 9 summarizes body location combination(s) that achieved a CCR greater than 90% while walking on different walking surfaces. There are total two, six, twelve, and four walk patterns with respective combinations of two, three, four, and five body locations that yielded a CCR greater than 90% for all the seven walking surfaces. Also, the minimum number of body locations required in walk patterns to achieve a CCR greater than 90% was two for flat uneven and banked left surfaces, and one for banked right, stair up, stair down, slope up and slope down surfaces.

Classification performances of the best body location combinations are presented in Tables 4 and 5. Most importantly, a minimum of two IMUs at two body locations such as the left shank and right shank or the right thigh and right shank are needed to classify walk patterns with a CCR greater than 90% for all the seven walking surfaces, respectively, and therefore, these body location combinations are considered to be the best body location combinations for the classification of walk patterns of overweight individuals walking on real-world irregular surfaces. In addition, data from IMUs attached to the left shank and right shank and IMUs attached to the right thigh and right shank achieved a CA (mean (± SD)) of 97.24 (± 2.17)% and 95.87 (± 4.02)%, respectively. Moreover, IMUs attached to the left shank and right shank can classify walk patterns over various surfaces with a CCR (mean (± SD)) of 95.52 (± 3.76)% for flat uneven, 95 (± 3.63)% for banked left, 95.70 (± 3.05)% for banked right, 99.56(± 0.29)% for stair up, 99.04 (± 0.28)% for stair down, 100 (± 0)% for slope up and 95.88(± 1.64)% for slope down surfaces. Furthermore, IMUs attached to the right thigh and right shank can classify walk patterns over various surfaces with a CCR (mean (± SD)) of 90.35 (± 7.45)% for flat uneven, 90.61 (± 8.99)% for banked left, 95.88 (± 3.54)% for banked right, 99.56 (± 0.29)% for stair up, 98.77 (± 0.65)% for stair down, 100 (± 0)% for slope up and 95.96 (± 1.5)% for slope down surfaces.

**Table 4:** Walk pattern classification performance for the combination of right shank and left shank.

Mean ± SD classification accuracy (CA) (%)	Mean ± SD correct classification rate (%) for different walking surfaces						
	Flat uneven	Banked left	Banked right	Stair up	Stair down	Slope up	Slope down
97.24 (± 2.17)	95.52 (± 3.76)	95 (± 3.63)	95.70 (± 3.05)	99.56 (± 0.29)	99.04 (± 0.28)	100 (± 0.0)	95.88 (± 1.64)

**Table 5:** Walk pattern classification performance for the combination of right thigh and right shank.

Mean ± SD classification accuracy (%)	Mean ± SD correct classification rate (%) for different walking surfaces						
	Flat uneven	Banked left	Banked right	Stair up	Stair down	Slope up	Slope down
95.87 (± 4.02)	90.35 (± 7.45)	90.61 (± 8.99)	95.88 (± 3.54)	99.56 (± 0.29)	98.77 (± 0.65)	100 (± 0.0)	95.96 (± 1.5)

## Discussion

The objective of this study was to identify the minimum number of body locations for the recording of IMU data that could characterize the walk patterns of overweight individuals over irregular walking surfaces. To achieve this goal, we investigated two criteria: (i) whether the walk patterns over various irregular surfaces could exhibit significant variation using statistical analysis and (ii) whether the walk patterns over various irregular surfaces could be classified with a CCR > 90% using a deep learning method. The findings of this study demonstrate that a minimum of two IMUs need to be attached on either one or both of the legs for the classification of the walk patterns of overweight individuals while walking on a range of real-world irregular walking surfaces with a CCR greater than 90% for each surface.

IMU data from at least two body location combinations, namely, (i) the right shank and left shank and (ii) the right thigh and right shank, were able to classify the walk patterns of overweight individuals on various real-world walking surfaces individually with a creditable classification performance (i.e., CCR > 90%) (Tables 4 and 5). In addition, the walk patterns defined using IMU data from these two combinations achieved a CA (mean (± SD)) of 97.24 (± 2.17)% and 95.87 (± 4.02)% respectively, and these values are very close to the highest CA (mean (± SD) of 97.78 (± 1.72)%) obtained by exploring all possible body location combinations (Fig. 6, Tables 4 and 5). These findings imply that attaching a large number of IMUs is unnecessary because a minimum of two IMUs can provide adequate variation in the walk patterns of overweight individuals for the classification of various irregular walking surfaces. Our results are in agreement with earlier research^20^, which suggests that increasing the number of IMUs might slightly improve the overall CA but is limited by the burden of wearing additional wearable devices. Furthermore, different individuals (such as middle-aged healthy women and Parkinson’s disease patients) choose the shank and ankle as their most preferred body locations because it is comfortable to wear IMUs hidden under clothes while using a lavatory^19,25^.

The characterization of walk patterns over all walking surfaces using data from a single IMU attached to a particular body location would be challenging. Data from both single and combinations of multiple IMUs were analyzed through ANOVA analysis to find whether only single IMU could enough to classify irregular walking surfaces. More specifically, data from a single IMU attached to a particular body location showed insignificant variation among surfaces (Fig. 4). In contrast, IMU data from a group of body locations while walking on a particular surface showed significant variations among body locations (Fig. 5), suggesting that a combination of body locations may result in greater variation in walk patterns, which might allow the classification of various walking surfaces. For example, IMU data from the trunk did not significantly differ across all surfaces (Fig. 4), and thus, compared with the trunk, other body locations provided data that showed more significant differences among surfaces (Fig. 5) might be combined with the trunk to increase the variation in walk patterns and improve the ability to classify various walking surfaces. Subsequently, data from the trunk alone achieved a CA greater than 90% (Fig. 6), but the trunk could classify only four surfaces (i.e., stair up, stair down, slope up and slope down) with a CCR greater than 90% (Fig. 9), which suggests that not the trunk alone but rather a combination of body locations may define walk patterns with greater variation that could be used to classify a variety of irregular walking surfaces with creditable classification performance. This finding of the current study is in agreement with previous studies^4,23^, which reported a low accuracy rate for the classification of various surfaces by employing only one body location.

Exploring all possible combinations of body locations is crucial for identifying the most appropriate locations from where IMU data should be collected to define walk patterns with the highest variations in irregular walking surfaces. In this study, we employed a simple technique^22^ to find all possible combinations of body locations as well as ANOVA analysis to reveal the variation in IMU data from each of the tested combinations. More specifically, the walk patterns from almost all combinations of body locations with the exception of the combination (wrist, right thigh and left shank) achieved a mean CA higher than 90% (Fig. 6). However, further investigation of the CCR (Figs. 7–9) revealed that only 24 combinations of body locations achieved a CCR greater than 90% for each of the seven walking surfaces (Fig. 9). This finding was in agreement with previous studies^4,22,23^, which demonstrated an inability to classify various real-world irregular walking surfaces with most of the combinations. We found that minimum two combinations among all tested body locations could characterize walk patterns with high variation and classify a variety of irregular walking with creditable classification performance at the same time (Tables 4 and 5). Thus, two combinations, namely, the right shank and left shank and the right thigh and right shank, are the most appropriate combinations of body locations for the attachment of IMUs in overweight individuals. Although wrist-worn wearable devices are commonly used, the IMU on wrist failed to show adequate data variation as well as classification accuracy across irregular surfaces (Fig. 4b and 6). One of the main reasons might be the arms’ swing pattern of overweight individuals exhibits range of motion lower than their counterparts^34,35^ and thereby is very similar regardless the nature of walking surfaces. This makes the IMU on wrist to be incompatible for the walking surface classification. Therefore, although wrist worn wearables have many other benefits (e.g., step count, heart monitoring), those might not be effective in identifying fall risk through AI applications.

The combination of both accelerometer and gyroscope data from an IMU is necessary to achieve greater variation in walk patterns, which makes it suitable for the classification of real-world irregular walking surfaces. More specifically, although variations in accelerometer data were more significant than those in gyroscope data across walking surfaces, both data types showed similar significant deviation across body locations (Figs. 4 and 5). This finding implies that the utilization of 3D gyroscope data along with 3D accelerometer data from an IMU attached to only one body location may not result in greater variation in walk patterns, but greater variation in walk patterns could be achieved by using a combination of body locations for the classification of real-world irregular surfaces. This findings is also consistent with previous studies in clinical settings^36–39^ and deep learning-based walk-pattern classification^4,23^.

It is difficult to compare our results with previous studies^4,23,24^ due to differences in the target group (i.e., participant profile and health condition), IMU numbers and locations, surfaces and model performance metrics among the studies. However, the classification results achieved for each real-world irregular surface using different body location combinations in the current study were in line with the classification results obtained in previous studies^4,23,24^. Similar to the previous studies, low classification accuracies were observed for similar planar surfaces (e.g., flat uneven and slightly banked) when compared to steep inclined and declined surfaces (e.g., staircase and slope) using most of the combinations of multiple body locations in current studies (Fig. 7, 8 and 9). The main reason could be the kinematic changes during steep incline/decline movement is greater than that on planar surfaces^10,13^. In addition, unlike any other previous studies^4,23,24^ the current study pinpointed two combinations of body locations (i.e., the right shank and left shank; and the right thigh and right shank) that could classify a range of real-world irregular surfaces with creditable accuracy result (i.e., CCR > 90%) (Table 4 and 5). We found that the shank, which was the location closest to the ground, resulted in the highest variance in walk patterns among surfaces^23^, which makes the combination of the right shank and left shank for the attachment of IMUs ideal for the classification of the walk patterns.

This study is notably different from previous studies and has significant findings in several contexts. Unlike previous studies^9,16,17,40–42^, walk patterns were defined using IMU data collected while walking on real-world irregular surfaces. Additionally, unlike previous studies^4,23,24^, the current study analyzed the variation in IMU data from different body locations and explored the walk pattern classification performance of all possible combinations of body locations to identify the most appropriate body locations for classification of the walk patterns of overweight individuals over irregular walking surfaces. Moreover, unlike any of the previous studies, the current study recommends using a minimum number of IMUs (i.e., only two IMUs at two body locations) for classification of the walk patterns of overweight individuals with creditable classification performance. Using only two IMUs (in two wearable devices) at two convenient body locations is more feasible in terms of daily walking in outdoor because it ensures more flexibility with less burden on overweight individuals^20^. While there is a common belief among the overweight population that nothing can be done to prevent falls^43,44^, these individuals intend to engage in fewer physical activities as one of the fall prevention strategies. However, two IMUs embedded into wearable devices could be used to improve the mental and physical strength of the targeted overweight population during the period of gait adaptability training to gain confidence of walking on irregular surfaces. Therefore, the findings from this study will potentially inform AI-based healthcare applications that can be implemented in wearable devices to minimize the risk of falling in vulnerable individuals.

Although the current study has great potential, some limitations also need to be mentioned. For instance, this study included only ten adult overweight participants aged from 19 to 33 years, but the subjects had no athletic or physical workout experience. A few potential body locations, such as the neck, thorax, and arm, were not tested. However, the neck and thorax are kinematically stable locations and might create walk patterns with minimal variation, whereas the arm was found to be the least preferred location in previous studies^19,25^. In addition, since wrist-worn wearable devices are most popular, we will conduct a comprehensive study on movement of wrist variation across normal-weight and overweight population over range of surfaces. The limitations of the current study provide directions for our future research. This study will be extended by including a substantial number of middle-aged and older individuals in the future. The findings of this study will aid the development of AI-based healthcare applications that can recognize a range of walking surfaces and be attached at user-friendly body locations to reduce the risk of potential falls among the overweight population.

## Conclusion

By leveraging the relationship between walking patterns and irregular surfaces, this study has identified the minimum number of body locations in overweight individuals suitable for attaching IMUs. It has been demonstrated that classifying irregular walking surfaces using a single IMU poses a challenge. Data recorded from at least two IMUs has the capability to accurately classify walking patterns while traversing most real-world irregular walking surfaces. Among all possible combinations of body locations, only two combinations stood out: the left shank and right shank, and the right thigh and right shank. These were identified as the most appropriate body locations for IMU attachment. The number and placement of IMUs discovered in this study hold the potential to aid the development of AI-based healthcare applications aimed at preventing falls among overweight individuals.

## Data Availability

The data generated and/or analysed for the current study are available from the publicly available database: Luo Y, Coppola SM, Dixon PC, Li S, Dennerlein JT, Hu B. A database of human gait performance on irregular and uneven surfaces collected by wearable sensors. Sci data. 2020;7(1):1–9. (https://doi.org/10.6084/m9.figshare.c.4892463 (accessed on 4 Nov 2021)).
